# Supplementary material for: Distribution and Health Risk Assessment of Trace Metals in Soils in the Golden Triangle of Southern Fujian Province, China
Source: Int J Environ Res Public Health. 2018 Dec 31;16(1):97. doi: 10.3390/ijerph16010097 (PMC6339116; doi:10.3390/ijerph16010097)
Supplement: Supplementary file 1 [file ijerph-16-00097-s001.pdf]

Supplementary Materials

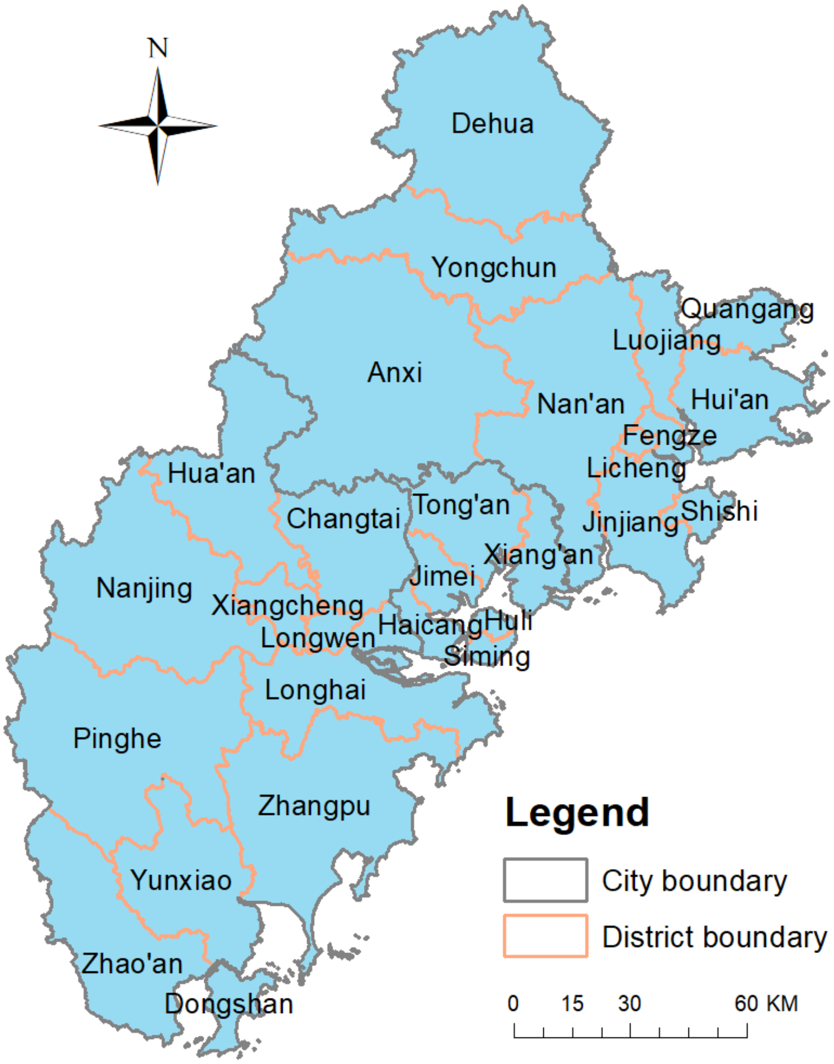

Figure S1. The 28 districts in the Golden Triangle of Southern Fujian Province.

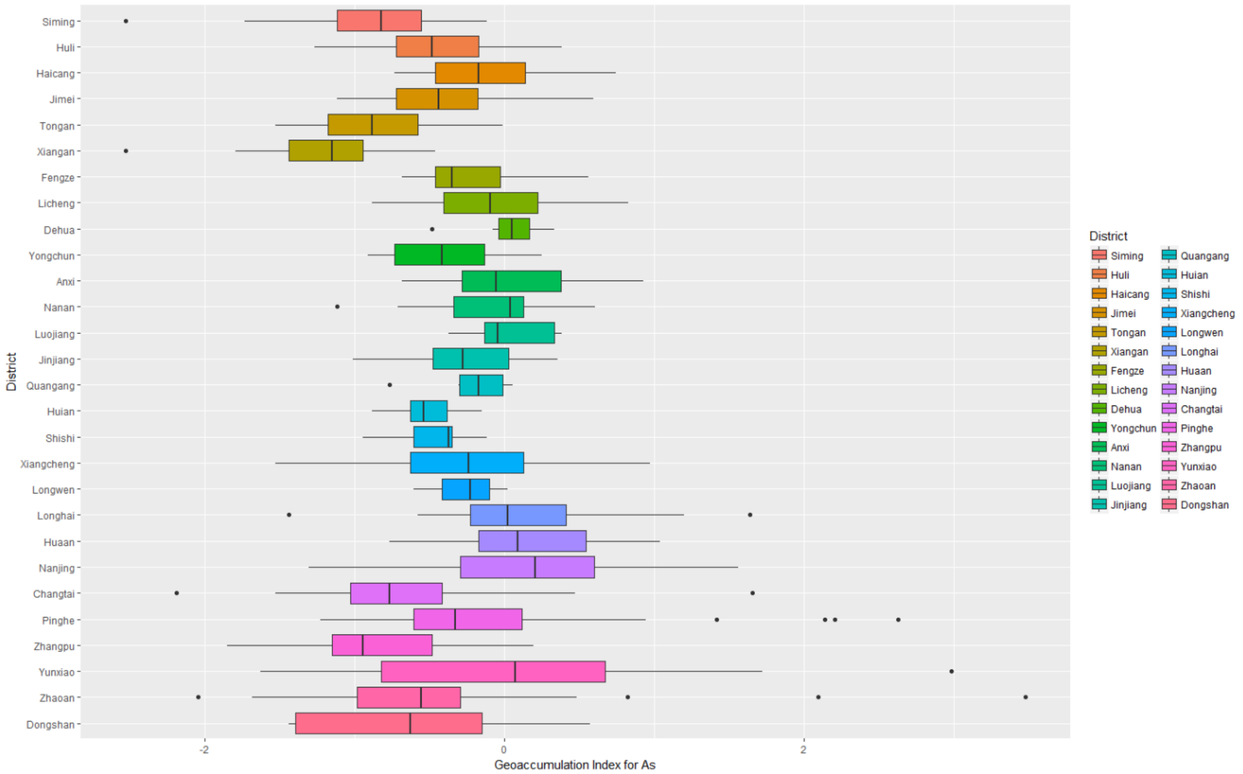
Siming
Huli
Haicang
Jimei
Tongan
Xiangan
Fengze
Licheng
Dehua
Yongchun
Anxi
Nanan
Luojiang
Jinjiang
Quangang
Huian
Shishi
Xiangcheng
Longwen
Longhai
Huaan
Nanjing
Changtai
Pinghe
Zhangpu
Yunxiao
Zhaoan
Dongshan
District
-2
0
2
Geoaccumulation Index for As
District
Siming
Huli
Haicang
Jimei
Tongan
Xiangan
Fengze
Licheng
Dehua
Yongchun
Anxi
Nanan
Luojiang
Jinjiang
Quangang
Huian
Shishi
Xiangcheng
Longwen
Longhai
Huaan
Nanjing
Changtai
Pinghe
Zhangpu
Yunxiao
Zhaoan
Dongshan

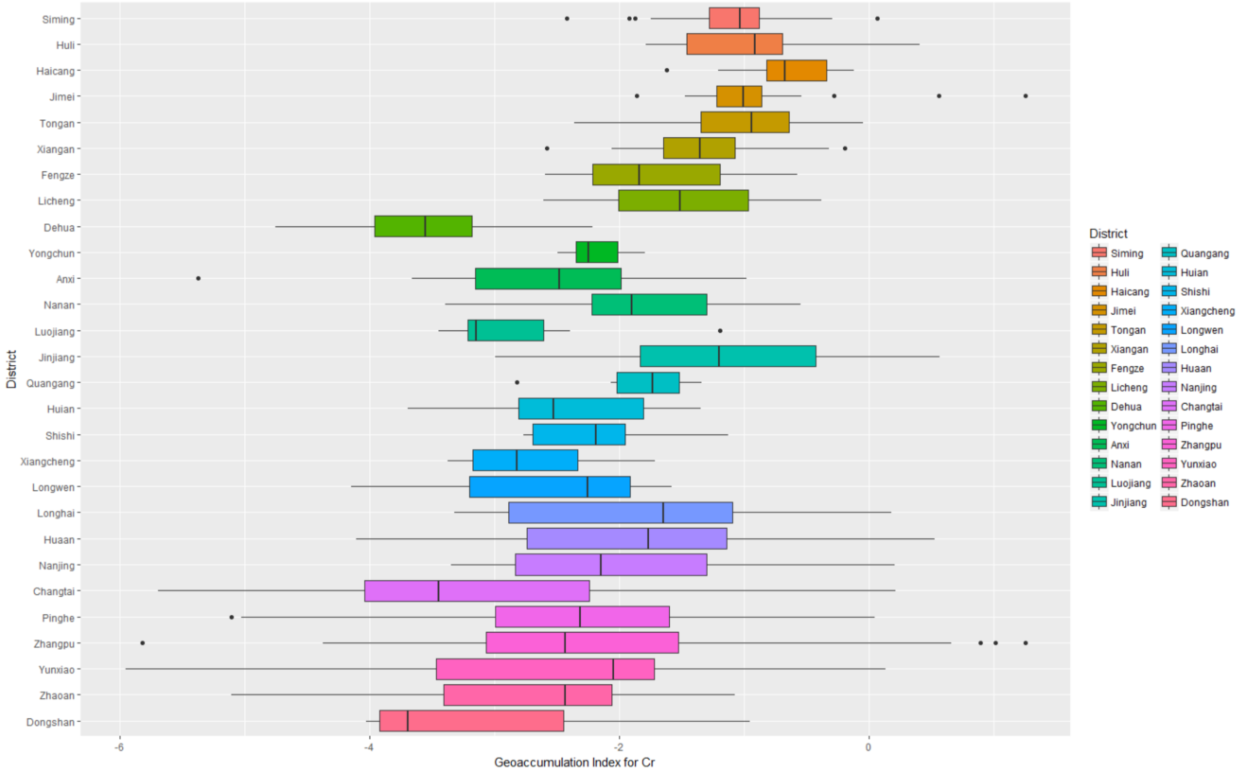
Siming
Huli
Haicang
Jimei
Tongan
Xiangan
Fengze
Licheng
Dehua
Yongchun
Anxi
Nanan
Luojiang
Jinjiang
Quangang
Huian
Shishi
Xiangcheng
Longwen
Longhai
Huaan
Nanjing
Changtai
Pinghe
Zhangpu
Yunxiao
Zhaoan
Dongshan
District
-6
-4
-2
0
Geoaccumulation Index for Cr
District
Siming
Huli
Haicang
Jimei
Tongan
Xiangan
Fengze
Licheng
Dehua
Yongchun
Anxi
Nanan
Luojiang
Jinjiang
Quangang
Huian
Shishi
Xiangcheng
Longwen
Longhai
Huaan
Nanjing
Changtai
Pinghe
Zhangpu
Yunxiao
Zhaoan
Dongshan

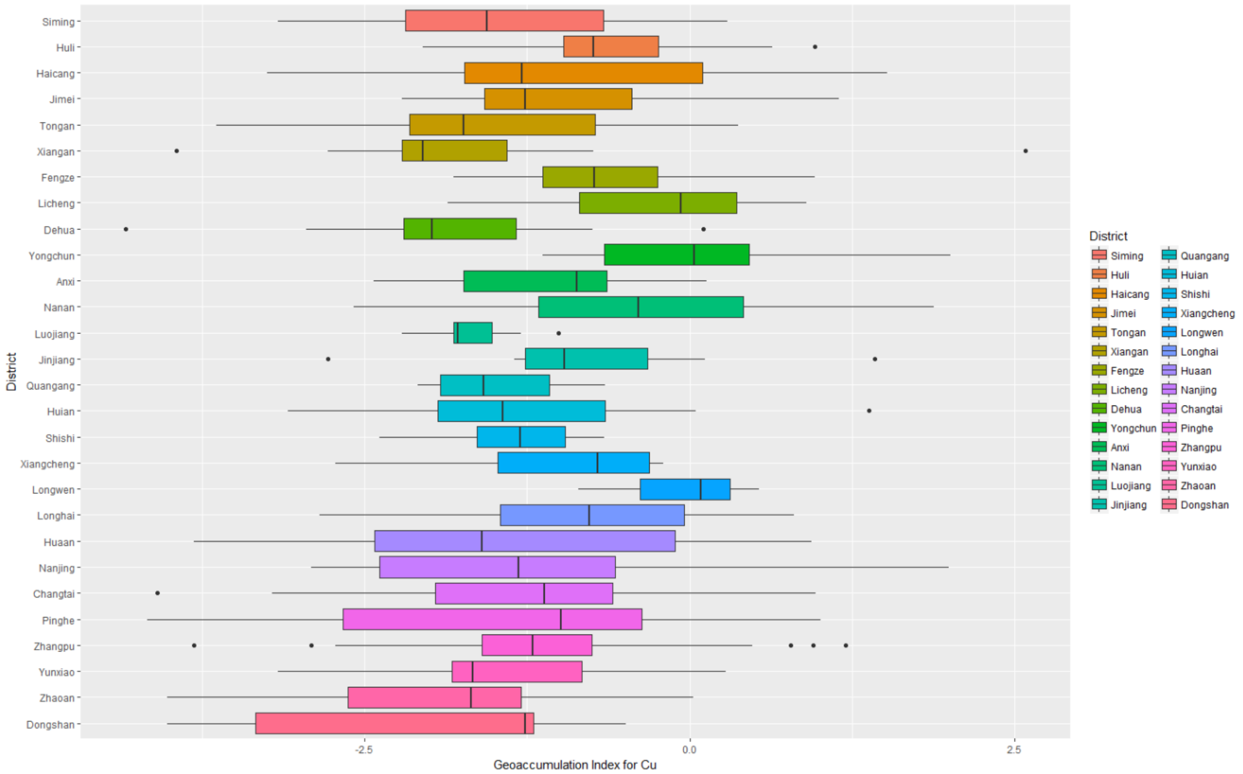

Siming
Huli
Haicang
Jimei
Tongan
Xiangan
Fengze
Licheng
Dehua
Yongchun
Anxi
Nanan
Luojiang
Jinjiang
Quangang
Huian
Shishi
Xiangcheng
Longwen
Longhai
Huaan
Nanjing
Changtai
Pinghe
Zhangpu
Yunxiao
Zhaoan
Dongshan
District
-2.5
0.0
2.5
Geoaccumulation Index for Cu
District

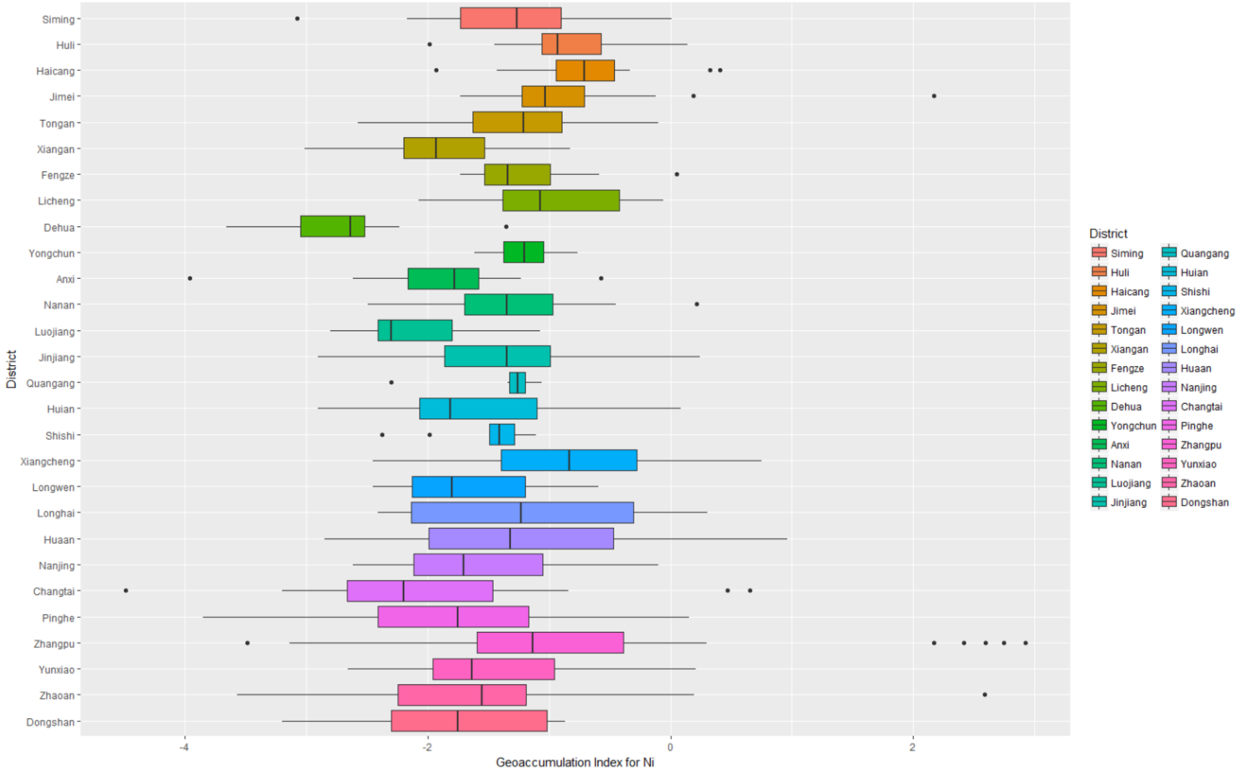

Siming
Huli
Haicang
Jimei
Tongan
Xiangan
Fengze
Licheng
Dehua
Yongchun
Anxi
Nanan
Luojiang
Jinjiang
Quangang
Huian
Shishi
Xiangcheng
Longwen
Longhai
Huaan
Nanjing
Changtai
Pinghe
Zhangpu
Yunxiao
Zhaoan
Dongshan
District
-4
-2
0
2
Geoaccumulation Index for Ni
District

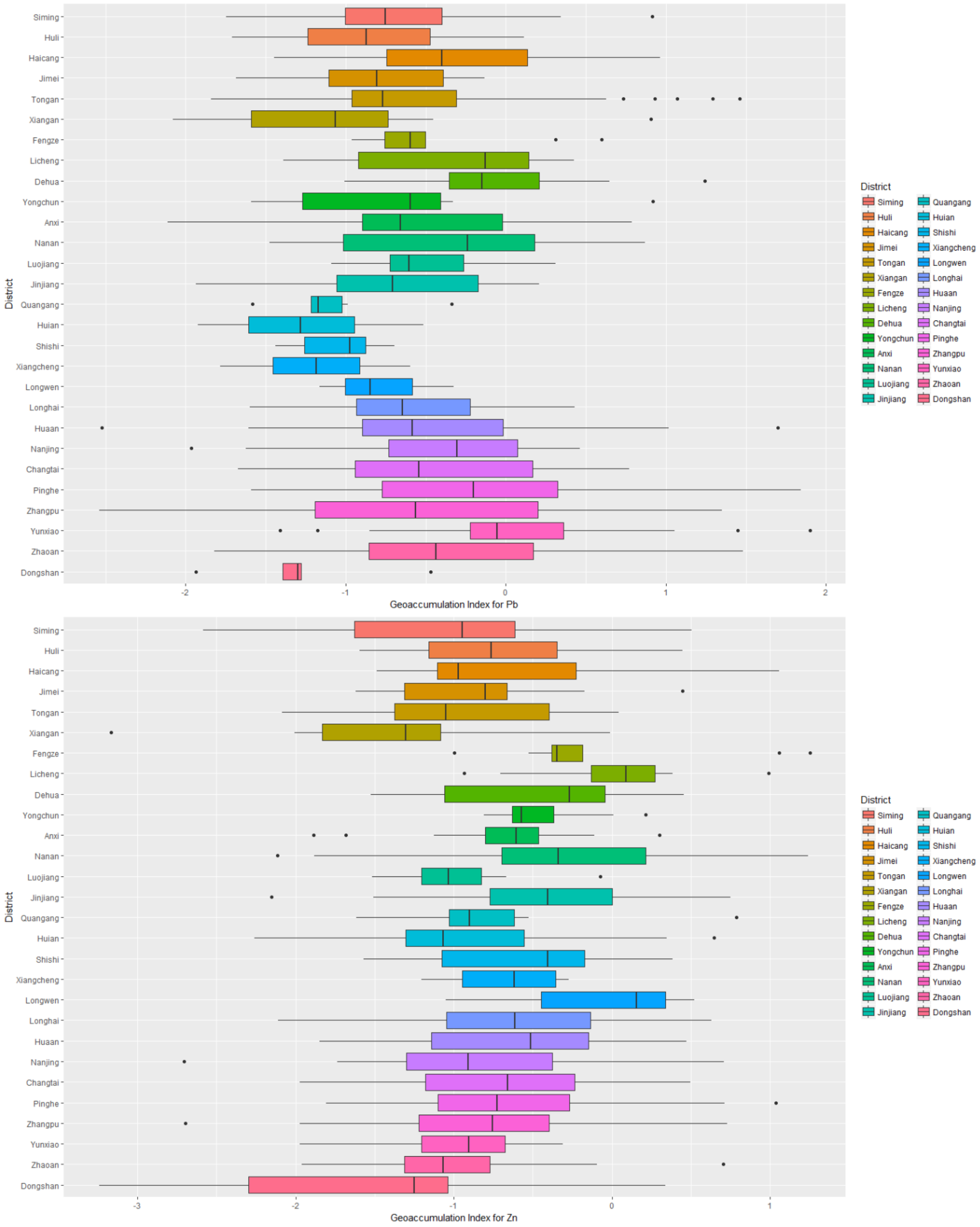

Figure S2. The I_{geo} values of soils in different districts of the Golden Triangle of Southern Fujian Province.

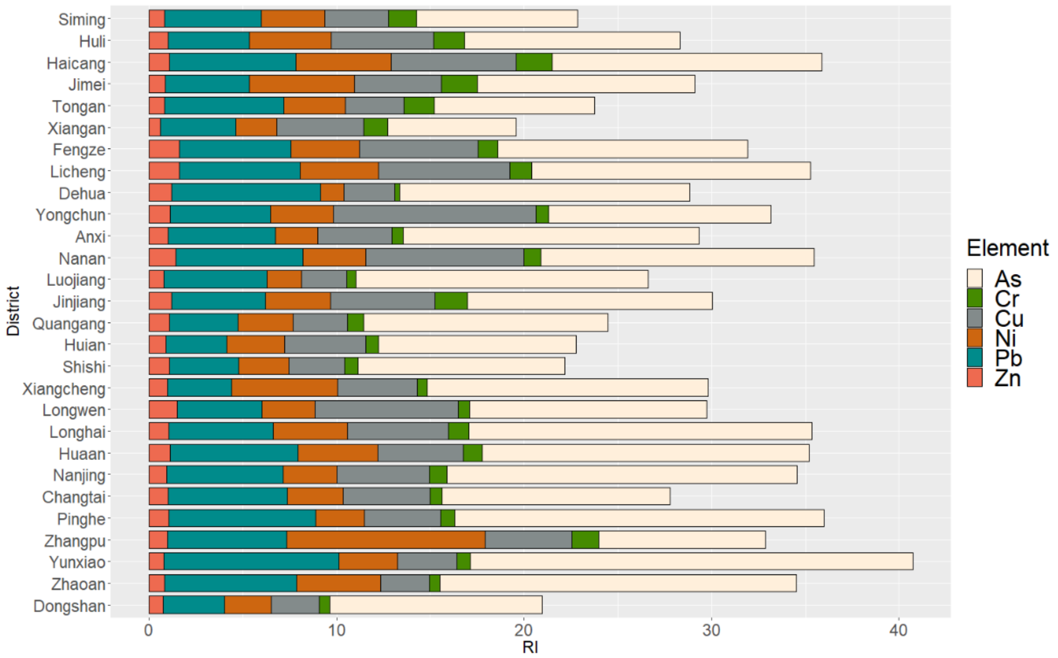

Figure S3. RI values of soils in different districts of the Golden Triangle of Southern Fujian Province.

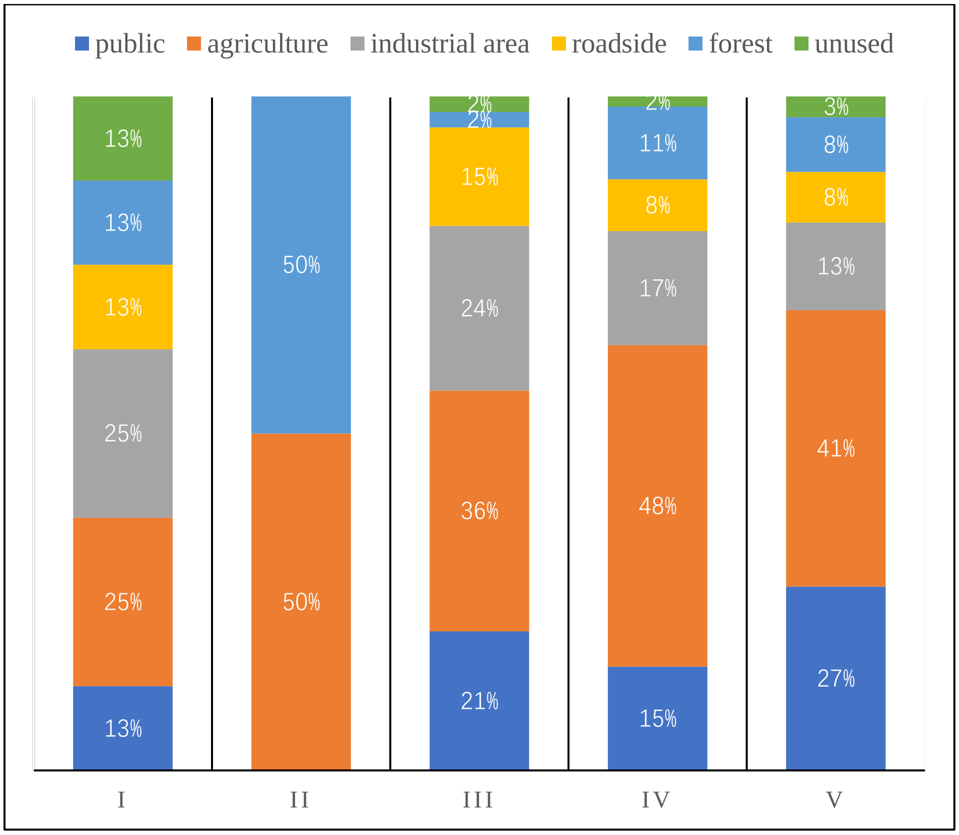

Figure S4. Land use types in groups I, II, III, IV, and V.

Table S1. Parameters description, values, and classification of I_{geo} and RI.

Method	Parameter	Description	Unit	Range	Level
I_{geo}	C_n	Content of the trace metal in the soil sample	mg/kg	<0	Unpolluted
				0–1	Unpolluted to moderately polluted
				1–2	Moderately polluted
				2–3	Moderately to Strongly polluted
	B_n	Background value of the specific trace metal	mg/kg	3–4	Strongly polluted
				4–5	Strongly to extremely polluted
				>5	Extremely polluted
RI	T_r	Toxic response factor		<150	Low
	C_i	Content of the trace metal in the soil sample	mg/kg	150-300	Moderate
	C_0	Background value of the specific trace metal	mg/kg	300-600	High
				>600	Serious

Table S2. Definitions and reference values for parameters used to estimate average daily intake (ADI).

Parameter	Description	Unit	Value	
C	concentration of trace metal in soil	mg/kg		
IR_{ing}	ingestion rate	mg/day	100 (adults)	200 (children)
IR_{inh}	inhalation rate	m^3/day	14.7 (adults)	7.63 (children)
CF	conversion factors	kg/mg	1×10^{-6}	
EF	exposure frequency	days/year	180	
ED	exposure duration	year	24 (adults)	6 (children)
PEF	dust emission factor	m^3/kg	1.36×10^9	
SA	exposed area through dermal contact	cm^2	5700 (adults)	2800 (children)
SAF	adherence factor	mg/cm^2	0.2	
ABS	dermal absorption factor		0.001	
BW	body weight	kg	57 (adults)	22 (children)
AT	average exposure time per year	days	365 × ED (noncarcinogens)	70 × 365 (carcinogens)

Table S3. Reference doses (RfD) and cancer slope factors (SF) for trace metals.

Item	Unit	As	Cr	Cu	Ni	Pb	Zn
RfD_{ing}	mg/(kg·d)	0.0003	0.003	0.04	0.02	0.0035	0.3
RfD_{inh}	mg/(kg·d)	0.0003	0.000029	0.0402	0.0206	0.00352	0.3
RfD_{derm}	mg/(kg·d)	0.000124	0.00006	0.012	0.0054	0.000525	0.06
Sf_{inh}	(kg·d)/mg	15.1	42		0.84		

Table S4. Descriptive statistics of trace metals in different district soils of the Golden (mg·kg^{-1}).

District	As (mg/kg) Mean ± SD	Cr (mg/kg) Mean ± SD	Cu (mg/kg) Mean ± SD	Ni (mg/kg) Mean ± SD	Pb (mg/kg) Mean ± SD	Zn (mg/kg) Mean±SD
Siming	4.97±1.6 b	31.07±11.26 ab	14.63±10.48 bc	9.19±4.35 b	35.88±17.07 b	68.62±35.49 b
Huli	6.63±1.86 ab	34.36±14.63 ab	23.64±12.79 abc	11.73±3.86 b	30.31±10.79 b	83.51±35.21 ab
Haicang	8.33±2.77 ab	39.68±11.1 a	28.73±30.96 abc	13.78±6.42 ab	46.92±24.25 ab	90.61±59.6 ab
Jimei	6.71±2.07 ab	39.79±30.64 a	19.98±16.11 abc	15.16±18.88 ab	31.11±9.13 b	72.07±30.57 ab
Tong'an	4.95±1.4 b	32.99±11.94 ab	13.53±9.92 c	8.89±3.54 b	44.24±32.94 ab	69.21±27.4 b
Xiang'an	3.96±1.18 b	26.86±11.6 abc	19.94±45.05 abc	5.92±2.33 b	27.96±19.67 b	51.00±24.02 b
Fengze	7.70±2.26 ab	22.31±11.44 abc	27.19±20.25 abc	9.94±4.75 b	41.43±18.06 ab	133.6±82.9 a
Licheng	8.59±3.08 ab	24.42±11.66 abc	30.28±16.43 abc	11.23±5.4 b	45.06±17.65 ab	133.71±50.49 a
Dehua	8.94±1.27 ab	6.06±3.23 c	11.56±9.53 c	3.43±1.76 b	55.38±29.09 ab	99.46±43.91 ab
Yongchun	6.86±1.9 ab	13.76±2.29 bc	46.69±40.51 a	9.01±1.71 b	37.42±24.64 ab	93.13±24.99 ab
Anxi	9.11±3.08 ab	12.31±7.64 c	17.18±9.35 bc	6.06±2.89 b	40.09±21.14 ab	82.91±30.76 ab
Nan'an	8.42±2.39 ab	19.03±9.52 abc	36.36±32.03 ab	9.04±4.76 b	47.29±23.95 ab	119.09±74.25 ab
Luojiang	9.01±1.7 ab	10.07±6.71 c	10.36±2.81 c	5.06±2.15 b	38.21±12.35 ab	66.18±22.45 b
Jinjiang	7.55±2.11 ab	35.58±26.6 ab	24.08±21.67 abc	9.37±6.03 b	34.93±14.03 b	100.19±50.03 ab
Quangang	7.53±1.45 ab	17.93±5.66 abc	12.47±5.15 c	7.93±1.97 b	25.72±8.21 b	88.77±63.09 ab
Hui'an	6.11±0.83 ab	13.41±6.38 bc	18.75±18.7 abc	8.37±5.64 b	22.45±6.62 b	75.18±41.96 ab
Shishi	6.38±1.05 ab	14.82±6.21 bc	12.86±4.79 c	7.22±1.71 b	25.68±4.73 b	91.03±39.19 ab
Xiangcheng	8.68±5.93 ab	10.70±5.82 c	18.48±10.55 abc	15.18±13.15 ab	24.05±8.26 b	80.03±22.85 ab
Longwen	7.30±1.55 ab	12.43±8.66 bc	33.07±14.55 abc	7.63±5.1 b	31.43±9.42 b	125.43±60.11 ab
Longhai	10.58±5.63 ab	22.44±17.06 abc	23.27±15.5 abc	10.74±6.39 b	38.7±15.01 ab	88.08±40.75 ab
Hua'an	10.07±3.74 ab	21.65±19.96 abc	19.67±18.17 abc	11.50±9.44 b	47.47±37.76 ab	93.06±43.12 ab
Nanjing	10.78±6.23 ab	19.53±15.84 abc	21.29±27.44 abc	7.75±4.43 b	43.51±16.49 ab	76.05±41.14 ab
Changtai	7.03±6.27 ab	13.74±19.27 bc	19.95±18.17 abc	8.01±9.28 b	44.47±24 ab	83.83±40.13 ab
Pinghe	11.38±11.4 ab	15.51±12.48 bc	17.63±14.8 bc	c7.05±5.11 b	54.46±34.45 ab	87.95±47.13 ab
Zhangpu	5.14±2.05 b	29.59±41.7 abc	20.01±18.29 abc	28.57±44.69 a	44.50±31.07 ab	80.73±42.19 ab
Yunxiao	13.65±15.8 a	14.76±15.31 bc	13.59±9.36 bc	8.46±4.92 b	65.16±45.03 a	64.87±20.41 b
Zhao'an	11.00±19.51 ab	11.48±6.93 c	11.23±8.22 c	12.11±23.88 b	49.03±32.82 ab	69.68±36.07 b
Dongshan	6.56±4.02 ab	11.24±12.07 c	11.16±8.68 c	6.68±3.8 b	22.86±8.94 b	61.52±56.64 b

* For each column, a significant difference at the $p < 0.05$ level is indicated by different letters (a, b, and c).

Table S5. Average concentrations (mg/kg) of heavy metals in the soils of different sampling site groups.

**Group**	**As**	**Cr**	**Cu**	**Ni**	**Pb**	**Zn**
I	4.63±1.53c	103.95±43.89a	54.95±16.41a	107.95±34.91a	23.98±14.76c	88.1±39.95ab
II	45.8±26.3a	27.35±25.19b	20.25±15.13bc	10.98±10.02bc	41.45±11.19bc	63.01±21.06b
III	9.01±4.47b	27.08±14.93b	28.87±23.9b	11.60±5.17b	44.01±25.21b	103.33±50.1a
IV	7.63±2.69bc	11.48±9.33b	9.8±5.06c	5.4±3.06c	66.12±36.64a	77.59±27.88b
V	5.03±1.81c	17.45±13.86b	10.07±6.77c	6.24±3.35c	28.05±10.03c	58.85±27.31b

* For each column, the different letters indicate a significant difference at the $p < 0.05$ level.
